# Using Baidu Search Engine to Monitor AIDS Epidemics Inform for Targeted intervention of HIV/AIDS in China

**DOI:** 10.1038/s41598-018-35685-w

**Published:** 2019-01-23

**Authors:** Kang Li, Meiliang Liu, Yi Feng, Chuanyi Ning, Weidong Ou, Jia Sun, Wudi Wei, Hao Liang, Yiming Shao

**Affiliations:** 1 Guangxi Key Laboratory of AIDS Prevention and Treatment & Guangxi Universities Key Laboratory of Prevention and Control of Highly Prevalent Disease, School of Public Health, Guangxi Medical University, Nanning, Guangxi China; 2 State Key Laboratory for Infectious Disease Prevention and Control, National Center for AIDS/STD Control and Prevention, Chinese Center for Disease Control and Prevention, Beijing, China

## Abstract

China’s reported cases of Human Immunodeficiency Virus (HIV) and AIDS increased from over 50000 in 2011 to more than 130000 in 2017, while AIDS related search indices on Baidu from 2.1 million to 3.7 million in the same time periods. In China, people seek AIDS related knowledge from Baidu which one of the world’s largest search engine. We study the relationship of national HIV surveillance data with the Baidu index (BDI) and use it to monitor AIDS epidemic and inform targeted intervention. After screening keywords and making index composition, we used seasonal autoregressive integrated moving average (ARIMA) modeling. The most correlated search engine query data was obtained by using ARIMA with external variables (ARIMAX) model for epidemic prediction. A significant correlation between monthly HIV/AIDS report cases and Baidu Composite Index (*r* = 0.845, *P* < 0.001) was observed using time series plot. Compared with the ARIMA model based on AIDS surveillance data, the ARIMAX model with Baidu Composite Index had the minimal an Akaike information criterion (AIC, 839.42) and the most exact prediction (MAPE of 6.11%). We showed that there are close correlations of the same trends between BDI and HIV/AIDS reports cases for both increasing and decreasing AIDS epidemic. Therefore, the Baidu search query data may be a good useful indicator for reliably monitoring and predicting HIV/AIDS epidemic in China.

## Introduction

Since the first AIDS case was reported in 1981, the virus has caused more than 60 million infections and more than 30 million deaths worldwide, making it the greatest threat to human health^1^. The severity of the AIDS epidemic and its perniciousness is also a social problem that needs an urgent solution. In recent years, the epidemic pattern of HIV/AIDS in China has gradually diversified and the scope of surveillance is continuously expanding. The traditional way of monitoring disease is to count the number of cases over a period of time by setting up sentinel hospitals throughout the country. A more modern way of monitoring diseases is to access the monitoring data that is released by the health department after sorting out the statistics. This method is collected by artificial means, covering a large area and for a period of time, which usually takes a lot of manpower, materials and time. Moreover, some HIV-infected patients may not be willing to report their symptoms to a doctor because of private issues and official data are usually released after two weeks of reported illness^2^. This lag in AIDS data collection and reporting is an impediment to the control of the epidemic.

The Internet search engine has become an important platform for public access to information as well as data archive, with the latter serving as research source in various disciplines. Research objects based on Internet data have not only been developed in the fields of economy, finance, and marketing such as products sales, trend of gas prices and stock market^3–5^, but also in medical research and epidemic analysis of infectious diseases. It can provide health authorities with important information regarding the emergence and spread of diseases in the city to complement traditional epidemic surveillance systems^6^. Google Trends was used to obtain a global search of Ebola during the Ebola outbreak from November 1st to December 27th, 2014^7^, and applied provided real-time tracking of the flu outbreaks^8–11^. Data from different search engines have also been utilized in tracking diseases such as malaria and breast cancer successfully^12,13^. Moreover, the real-time monitoring of disease trends using the Internet-based Google Insights has been explored on dengue fever in Singapore and Bangkok from 2004 to 2011^14^. Diseases such as AIDS, due to a variety of reasons, patients often remain silent in front of relatives, friends and even doctors about their own status, and turn to self-diagnosis through Internet searches. While providing convenience, it also has the advantage of user private information confidentiality. There is little research on Internet data analysis for HIV/AIDS in China, with biological indicators, treatment methods, epidemiology and demographic indicators principally applied to evaluate and monitor the local epidemics^15,16^. The Baidu index launched by the Baidu Inc is the main Internet search tool used in China. In March 2018, its market share in China was 73.02% which is far higher than search engine markets such as Google^17,18^. We therefore use the internet search data provided by the Baidu index to survey the HIV/AIDS epidemics in China.

We compare the internet data to the number of conventional epidemic reports in the same period to determine the relevance of Internet search behavior. Spatial difference analysis of the data is also performed to find the differences in spatial distribution of the AIDS-related search data. Lastly, we developed an Autoregressive Integrated Moving Average with Exogenous Variables (ARIMAX) model based on the keyword search index of the Internet and examined whether it improved the model’s forecasting ability^19^. Our method will provide new reference approaches for the analysis, monitoring, and prevention of the AIDS epidemic.

## Result

### Descriptive analysis

**Figure 1:**
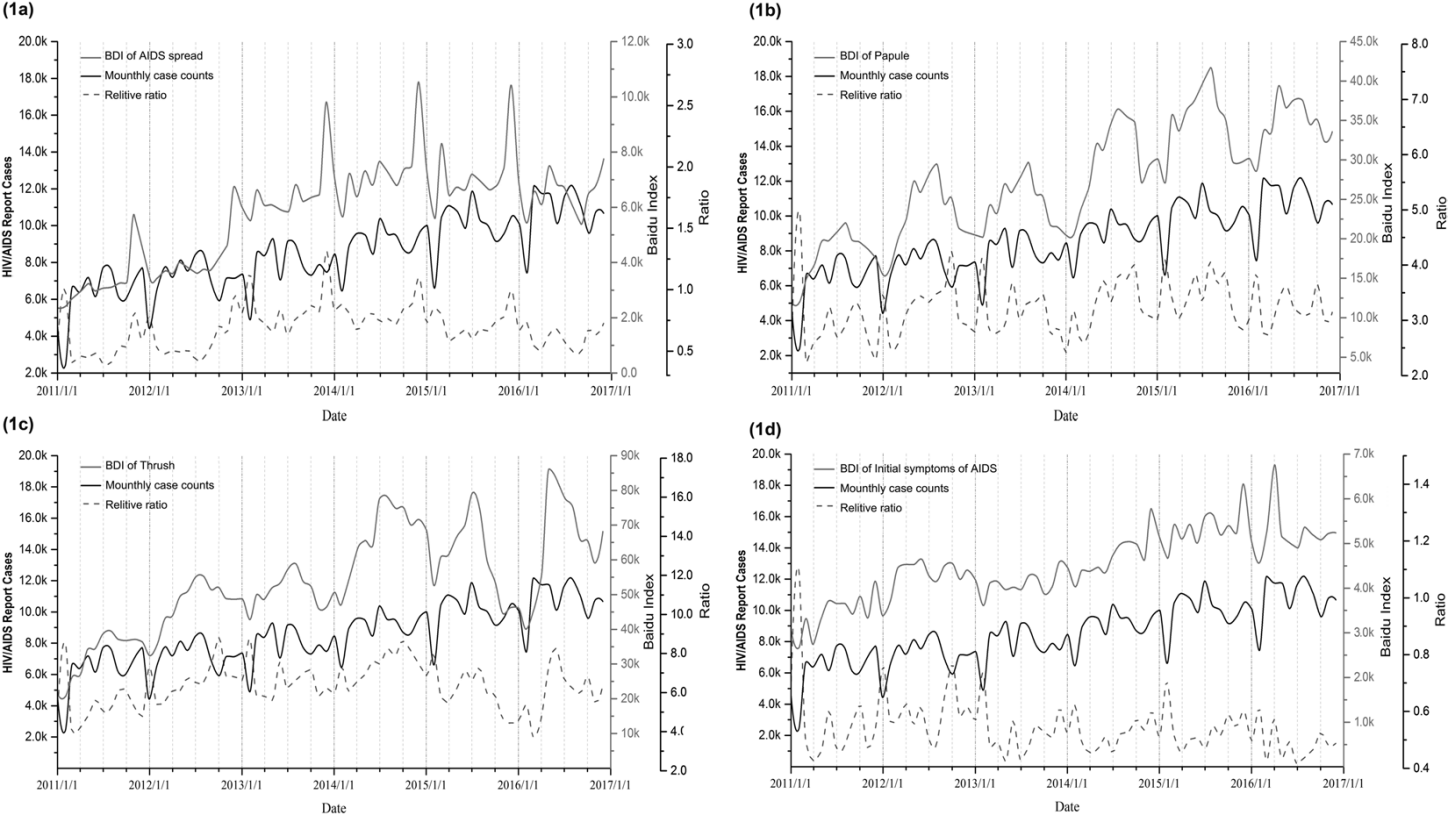
Time series of Some Keywords Search Index and monthly reported cases for HIV/AIDS in China, 2011–2016. This picture shows the time-series comparison curve between the Baidu search index and the national monthly report case number for the four keywords “AIDS spread,” “pimple,” “thrush,” and “Initial symptoms of HIV”. (The X-axis date interval is month. The Y-axis uses three coordinates, which the black Y-axis shows the number of monthly report cases, the red Y axis is the Baidu search index of the keywords and the blue Y-axis is the ratio of the search index to the monthly report cases); BDI: Baidu Search index.

This describes the parameters and tools used in this study, including the Baidu search engine and its daily search amounts and wide coverage to all of China, Baidu search index, etc. what does Fig. 1 try to analyze? The AIDS related search statistics are summarized in Table 1, which is conducted using Baidu search index data from January 2011 and June 2017 in China. Among the five categories of 50 keywords, we found that the keyword “AIDS”, “Prostitute”, “Short version for Gay”, “HIV” and “Sexual services” had the highest average index of search per month, and the keyword “Snow-mouth disease”, “AIDS testing Center”, “Best testing time for AIDS”, “AIDS-testing” and “AIDS low fever” search index was at a relatively low level. Besides, keywords in the Etiology and General category of AIDS had higher search index than other categories.

**Table 1:** The monthly search statistics of Baidu search index with AIDS related keywords from January 2011 to June 2017.

Categories	Search Keywords (in Chinese)	Search Keywords (in English)	Search Amount Mean ±SD	Minimum	Median	Maximum
General		AIDS	282794.76±81806.11	105084	287680	478888
		AIDS infection	3969.22±536.76	2697	3999	5600
		AIDS virus	7474.72±1450.09	4704	7300.5	11130
	HIV	HIV	80804.25±36113.52	26208	81807	201779
Epidemiology		Gay	70612.92±34610.10	31496	63577	227478
		Short version for Gay	95150.82±27032.44	56880	89094	196320
	MSM	MSM	18313.65±7210.63	11060	15177	43740
		Skating poison	31739.19±10564.72	13832	31031	48690
		Taking drugs	44442.03±22981.59	19065	40515	200105
		HIV/AIDS transmission route	50743.35±21089.82	15204	47318	103170
		Transmission of AIDS	7494.35±2175.75	4984	6960	18445
		Spread of AIDS	5761.00±1781.26	2356	6165	11563
		Street walker	49207.99±19012.56	25530	44356	103571
		Underground prostitute	8198.35±1512.05	6020	7828	15035
		Hotel prostitute	12688.99±4091.40	6960	12405	37386
		Guesthouse prostitute	12606.51±5170.44	5700	10703	26195
		Prostitute	186255.93±79940.38	109709	164874	655929
		Sexual services	63012.64±43128.26	6417	74734	159371
		Sauna Service	53452.07±11478.20	35616	54507	110732
		Male comrades	5889.83±2448.54	2940	5356	13560
		Gay website	197372.31±303577.34	33420	57195	1142400
		Short version for Gay website	41340.38±15108.44	16895	37433	90086
		Anal intercourse	19588.61±6819.02	10013	17458	34813
Diagnosis		AIDS detection	33018.03±9039.65	15428	31545	60729
		AIDS examination	8498.89±2372.57	5270	7859	17453
		AIDS-testing	3442.53±3376.01	217	2409	14911
		AIDS test strip	16544.07±6882.37	8525	14264	34348
		Best testing time for AIDS	2251.10±1388.90	60	2502	6386
		How to check AIDS	9682.33±4949.57	4216	7730	26629
		AIDS self-testing	10714.46±11162.65	1302	4852	43260
		AIDS testing Center	2845.25±1247.76	341	2589	5460
	HIV	HIV testing	11223.13±3308.57	6804	10095	25482
	HIV	HIV test strip	5470.21±3148.79	3150	4559	18368
		AIDS incubation period	34965.79±23678.05	12270	28222	155744
		AIDS window period	29875.68±13916.84	11718	26585	75609
AIDS Symptom		AIDS Symptoms	56036.86±34095.86	29120	52082	286564
		Initial symptoms of AIDS	24792.63±28036.33	6468	13037	135690
		Symptoms in AIDS window period	13617.51±3359.92	9780	12726	23580
		Early symptoms of AIDS infection	3664.33±1262.98	682	3658	9672
AIDS Symptom	HIV	Initial symptoms of HIV	4479.74±749.24	2660	4485	6750
		Papule	28113.04±7560.40	11935	27885	42160
		Thrush	55245.08±16240.63	20608	53695	98220
		Snow-mouth disease	3412.49±645.11	1288	3446	5301
		AIDS Diarrhea	4242.1±690.90	2688	4402	5890
		AIDS low fever	2112.93±609.71	961	2046	3999
		What are the symptoms of AIDS	12427.42±3307.87	6665	12302	21948
AIDS Treatment		AIDS treatment	10377.01±3175.92	7192	9989	30566
		Acyclovir	41032.24±17393.68	15820	39901	109585
		Zidovudine	5543.51±3237.67	1008	5730	24242
		Lamivudine	15011.61±2749.59	9641	14632	22041

Internet user search messages in Baidu using Chinese and the translation of each Chinese keywords is listed in English.

### Correlation analysis

Spearman’s rank analysis on the correlation between Baidu Search index and HIV/AIDS reported cases found that 11 out of the 50 keywords are not related to epidemic, 20 keywords have weak sequential time-series correlation. Only 19 search keywords have significantly correlated to the reported cases, of which correlation coefficient is greater than 0.6 (Table 2). We then illustrated the time-series comparison curve between the Baidu index of several keywords and the monthly number of reported cases in China. As Fig. 1 shows, although the four keywords have different search frequencies and relative ratios, their trends are the same, which increased or decreased with the search index rising and falling phenomenon.

**Table 2:** Correlation analysis of Baidu Search index and HIV/AIDS reported cases.

Keyword (in English)	Coefficients	*P* value	Keyword (in English)	Coefficients	*P* value
AIDS-testing	0.639	<0.001	Thrush	0.766	<0.001
AIDS virus	0.652	<0.001	Zidovudine	0.767	<0.001
AIDS test strip	0.654	<0.001	Street walker	0.771	<0.001
AIDS	0.662	<0.001	Acyclovir	0.776	<0.001
AIDS examination	0.665	<0.001	Initial symptoms of HIV	0.792	<0.001
AIDS incubation period	0.667	<0.001	How to check AIDS	0.799	<0.001
Taking drugs	0.700	<0.001	Prostitute	0.804	<0.001
Spread of AIDS	0.704	<0.001	HIV	0.819	<0.001
AIDS window period	0.730	<0.001	Papule	0.879	<0.001
Sexual services	0.751	<0.001			

Correlation coefficient is calculated by Spearman’s rank method. Only the key search keywords with correlation coefficients of 0.6 or above are listed.

### Cross-correlations analysis and composite index

**Figure 2:**
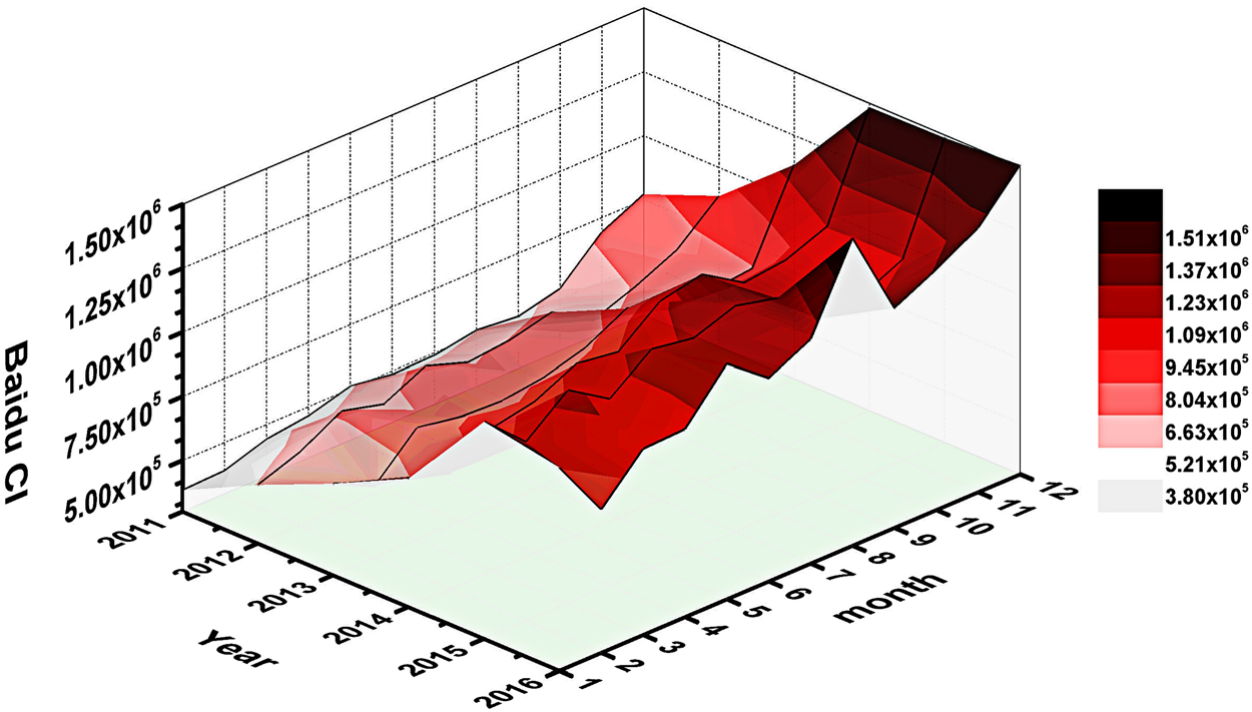
Time series of Baidu Composite Index in China from 2011 to 2016. This figure displays the three-dimensional changes in the year and month timescales of Baidu Composite Index from 1 January, 2011 and 31 December, 2016. (The X-axis date interval is month; the Y-axis time interval is year; the Z-axis is the national Baidu Composite Index (Baidu CI).

We carried out time-lapse correlation analysis of search keywords and obtained 17 keywords with the maximum cross-correlation coefficients above 0.5 in Table 3. We observed that the correlation progressively increases with the decrease in the days of lag, and then reaching a peak value at lag 0. Additionally, we calculate each keyword’s weight in the formula and add their Baidu index according to the weight to form the Baidu composite index. The Spearman correlation coefficient between monthly HIV/AIDS cases data and the composite index was 0.845 (*P* < 0.001). We also displayed AIDS related Baidu Composite Index (Baidu CI) trends in both annual and monthly time dimensions, and found a constant increase of it from 2011 to 2016 (Fig. 2). Moreover, The Baidu CI has two small and one big surge respectively in July, August and December of each year.

**Table 3:** Cross-correlation between monthly HIV/AIDS report cases and Baidu search index data.

Keyword (in English)	Maximum CCF	Lag (month)	*P* value	Keyword (in English)	Maximum CCF	Lag (month)	*P* value
Papule	0.875	0	<0.001	Zidovudine	0.638	0	<0.001
Early symptoms of HIV	0.781	0	<0.001	Acyclovir	0.620	0	<0.001
Thrush	0.777	0	<0.001	AIDS examination	0.615	0	<0.001
HIV	0.760	0	<0.001	AIDS virus	0.585	0	<0.001
Sexual services	0.746	0	<0.001	Prostitute	0.580	0	<0.001
AIDS window period	0.702	0	<0.001	AIDS-testing	0.578	0	<0.001
Spread of AIDS	0.678	0	<0.001	AIDS test strip	0.568	0	<0.001
Street walker	0.664	0	<0.001	AIDS	0.533	0	<0.001
How to check AIDS	0.657	0	<0.001				

CCF: Cross-Correlation Function.

### Regional analysis of correlation between search index and AIDS epidemic

**Figure 3:**
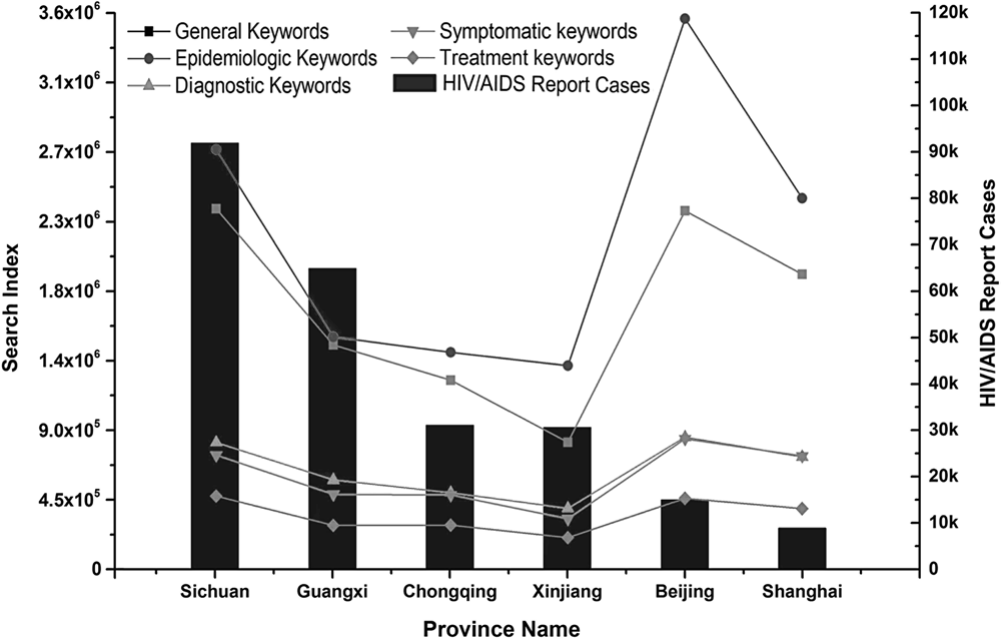
Comparisons of HIV/AIDS report cases and the five types of keywords in different provinces from 2011 to 2016. The column diagram shows the total number of HIV/AIDS report cases for six provinces; the line graph represents the five types of keywords total search index in each province.

AIDS epidemic in China has a big regional discrepancies among her 31 mainland provinces with 5 to 6 provinces reporting more than half of China’s total HIV/AIDS cases. We compared two provinces with very high epidemic (Sichuan, Guangxi), two provinces (Xinjiang and Chongqing) with middle level epidemic and two major cities (Beijing, Shanghai) with low level epidemic (Fig. 3). It is found that in the regions with high AIDS epidemic, the search index of various keywords is also relatively high. In contrast, in Beijing and Shanghai the number of HIV/AIDS cases is far lower than other provinces, but the search index of kinds of keywords is still at a relatively high level. General and epidemiologic keywords had a much higher search index than keywords related to diagnostic, symptomatic and treatment. This result was indicated, as individuals generally use keywords that are easy to comprehend and that are widely used.

**Figure 4:**
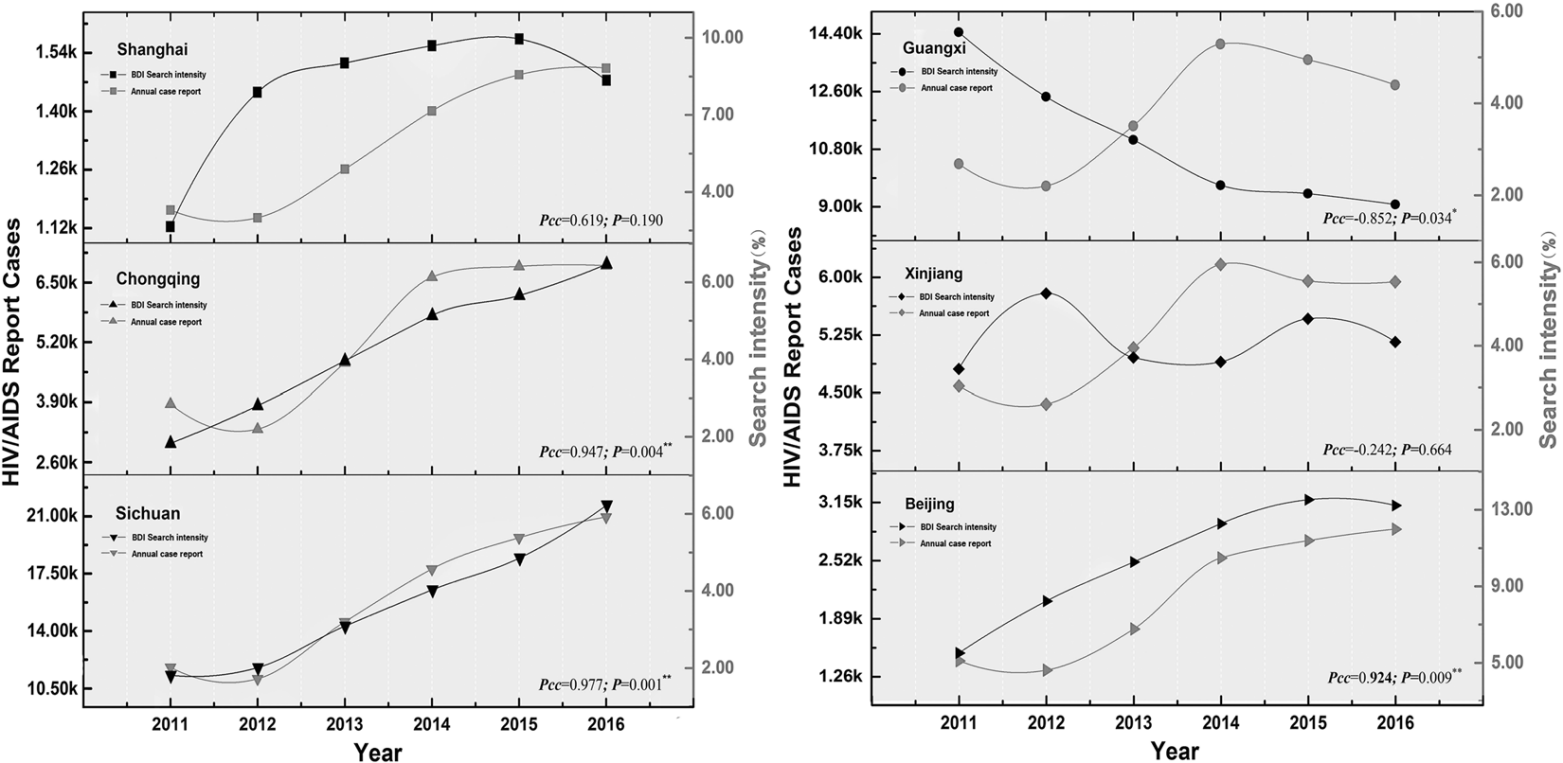
Search intensity and annual case counts. This figure describes the changes in annual case counts and the Web users Search intensity in different provinces from 2011 to 2016. The line charts represent the annual HIV/AIDS case counts (black), and Baidu Search intensity (gray) for all of the six provinces. Pcc: Pearson Correlation Coefficient.

Furthermore, we also found that there are significant differences in spatial distribution of the correlation between different keywords and the number of real cases. Although the search index for assorted keywords in Shanghai is high, the correlation between the search index and the number of HIV/AIDS is lower than that of Beijing and Sichuan provinces with the same keyword search volume. In Guangxi and Xinjiang province, on the one hand, the correlation between keyword searches and the number of AIDS cases was significantly lower than that of Sichuan and Chongqing province which had the same epidemic and search volume, and on the other hand, it showed a relatively weak negative correlation trend (Supplementary Fig. S1). We then graphed curves of annual HIV/AIDS case counts and internet users’ search intensity in each province from 2011 to 2016 (Fig. 4). Obviously, except for the downward trend in Guangxi and Xinjiang during 2015 to 2016, the intensity of search in other regions has been increasing year by year. The Internet search intensity is highest in Beijing and Shanghai, and the lowest in Sichuan province. The number of new cases of AIDS is increasing year by year in Sichuan and Chongqing, the overall decline of Guangxi and Xinjiang, and Shanghai and Beijing showed a stable trend. In terms of the correlation between search intensity and the number of AIDS cases, compared with other regions, Beijing, Sichuan and Chongqing are higher than that of other regions, which are above 0.9. This phenomenon implies that search behaviors related to AIDS vary throughout each province.

### Differencing and ARIMA model construction

The time dependence and seasonal change were found by the Supplementary Fig. S2. It can be seen that the two sequences show a significant upward trend and a tendency of fluctuating changes, suggesting that the sequence is not stable. The unit root test indicated that the sequence was non-stationary (the null hypothesis was that the sequence was non-stationary, P > 0.05).

The differential sequence y has trailing properties both in autocorrelation coefficient (ACF) and partial autocorrelation coefficient (PACF) (Supplementary Fig. S3a). According to the minimum information quantity AIC order criterion, the model ARIMA (0, 1, 2) (1, 0, 0)_12_ is finally fitted after several attempts. In addition, the parameter tests are all significant non-zero (Table 4, *P* < 0.05). Residual autocorrelation test shows that the residual series belong to the white noise sequence (Supplementary Fig. S3b).

**Table 4:** Characteristics of ARIMAX models: coefficients, standard errors, P value for coefficients and Ljung-Box test of residuals, MAPE, AIC.

Model	Variable	Parameter	Lag	Coefficients	Standard error	*P* value	Ljung-Box test	AIC	MAPE
Model 1	ARIMA	MA	1	0.939	0.117	<0.0001	0.269	1184.78	7.57%
		MA	2	−0.283	0.117	0.0185			
		SAR	12	0.779	0.098	<0.0001			
Model 2	ARIMA + Baidu CI	AR	1	−0.644	0.114	<0.0001	0.155	839.42	6.11%
		SMA	12	−0.100	0.292	0.0013			

ARIMA: autoregressive integrated moving average model, ARIMAX: ARIMA with external variables, AIC: Akaike information criterion, MAPE: mean absolute percentage error, MA: moving average, SAR: seasonal autoregressive, SMA: seasonal moving average.

### ARIMA model with external variables (ARIMAX)

**Figure 5:**
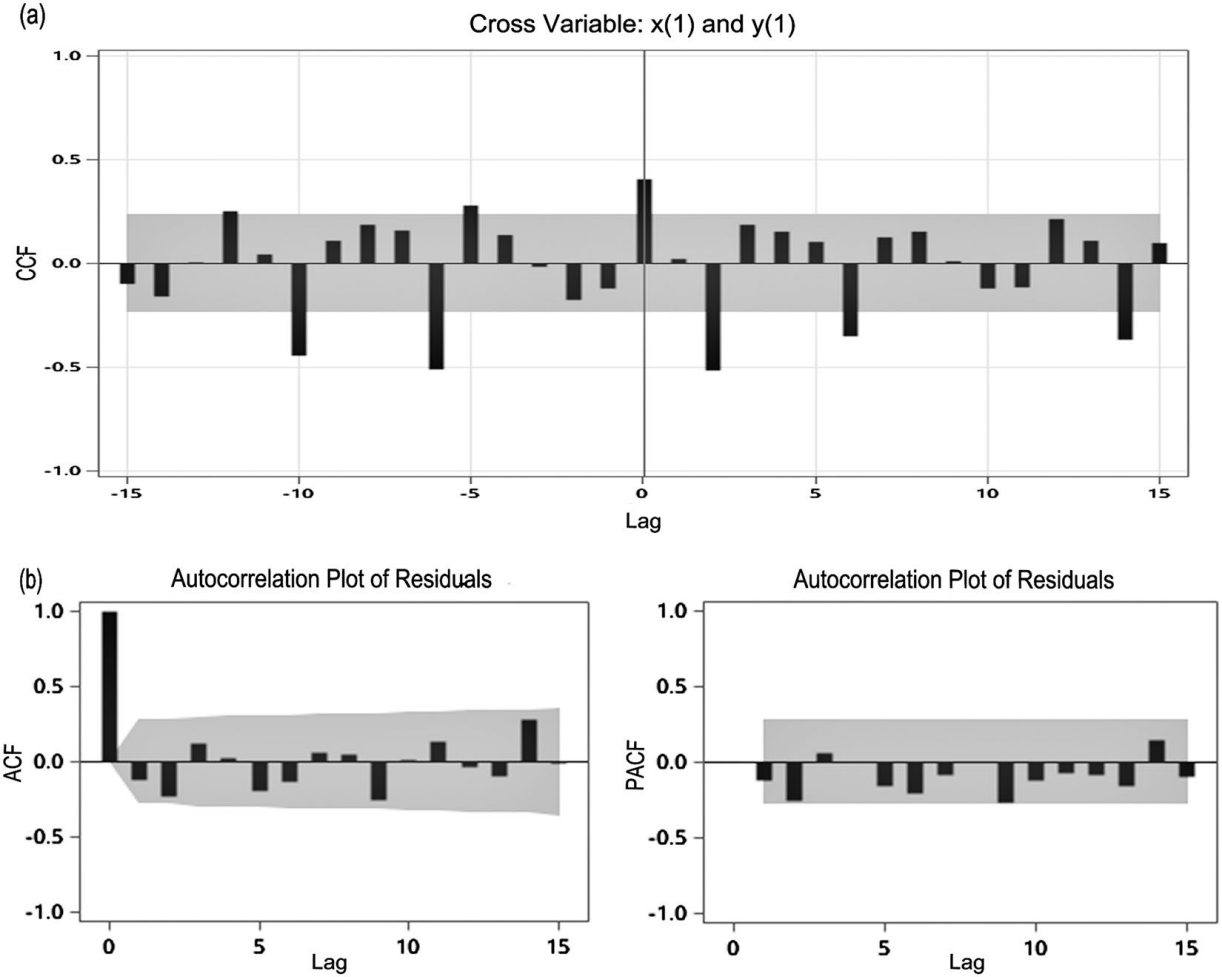
Autocorrelation check of residuals for the model, and the Interrelationships diagram of input sequence and output sequence. The X-axis gives the number of lags in weeks, the Y-axis is the value of the correlation coefficient, and the gray zone illustrate 95% confidence interval.

In the ARIMAX model, both the input sequence (x1_t_) and the output sequence (y1_t_) are stationary. Establishing co-correlation coefficient plots for different delay orders, we can see that differencing Baidu CI has a significant lag effect with differencing HIV/AIDS report cases and the parameters obtained after estimating the model are significantly non-zero, moreover, the residual autocorrelation test shows a random distribution that there is no autocorrelation in the residual (Table 4, Fig. 5). The Ljung-Box test of the residuals for all models could not reject the null hypothesis that the model exhibit significantly effective (P > 0.05).The ARIMAX model with BDI as predictor showed a better goodness of fit than the model without external variables (AIC = 839.42 vs 1184.78, respectively), and besides that the model including BDI showed a smaller MAPE than others for the forecast accuracy ((MAPE = 6.11%, the smaller the better (Table 4). The predicted cases of the final fit of the predictive model are basically consistent with the measured cases, which are within the 95% confidence interval (Supplementary Fig. S4).

## Discussion

The application of Internet data to the epidemic analysis of infectious diseases has become a hot topic in the prevention and control of infectious diseases and the online digital diseases surveillance tool based on Google Insight and Google Trends has been explored in recent years^10,20–22^. The official AIDS epidemic data in China is mainly generated by traditional monitoring systems in regions that lacked human and material resources, and where the regulatory system is less efficient^2^. This deficiency increased the risk of AIDS transmission and complexity of disease control. The detection system based on Internet search can avoid the delays and irregular reports of traditional detection systems^23,24^. Search engines, as query tools, can provide sensitive monitoring of disease and epidemic situation information before the diagnosis of the disease is reported, so as to improve the control of the disease. And the research on Internet Surveillance and Analysis Methods for Conventional Epidemics has achieved good development in various countries.

In this study, six categories of 50 related keyword search indexes were obtained from Baidu Index. Among them, the keywords searched for the general and epidemiologic categories were higher than other keywords, and the simple keywords such as “AIDS”, “Prostitute”, “HIV” and “AIDS Symptoms” were more frequently searched than other keywords. This is because people usually pay more attention to the vocabulary of AIDS etiologies and symptoms, and they are also good at searching with simple keywords. When cross-correlation analysis was performed to observe lag, no significant delays were observed in each keyword. The national Baidu composite index was then obtained by adding the sum of the weight of the filtered keywords. We found that the number of new AIDS cases correlate positively with the search index of various keywords. In other words, there is a significant temporal correlation between the Baidu search index and the actual epidemic. During the period when there are numerous cases, the search volume of relevant keywords increases, and then the Baidu index increases. After the epidemic has been alleviated and controlled, the Baidu Index fell. These phenomena demonstrated that the public’s attention to the epidemic developed along with the development of the epidemic^14,25^. Additionally, symptoms and treatment are keywords that have a strong correlation only with HIV-1 infected people and not the general population. These two keywords therefore are vital for using search engine data to monitor the AIDS epidemic.

In the overall trend of the Baidu Composite Index, there is a short-term surge in December of each year with no noticeable increase in new HIV infections in China. This may be associated with a large number of social media reports on World AIDS Day in the month of December. The increase awareness of the AIDS epidemics causes the surge in the search for AIDS-related information and the search index to inflate.

In regional difference analysis of Baidu Index, we find a positive correlation between the number of AIDS cases and the total distribution of various keywords in less developed provinces. This indicates that when the number of cases is large, the total amount of search volume is also large. However, although the AIDS epidemic in economically developed cities such as Beijing and Shanghai is much lower than other provinces and cities, the search index of various keywords is higher than that of the epidemic-higher provinces. In these developed regions, there may be a large amount of daily publicity and reporting by medical organizations and the media, and the overall cultural quality of the population is higher^26^. As a result, the number of search requests for the population increased, but the actual AIDS epidemic has not surged. In the correlation analysis, we found that in the regions where the AIDS epidemic has been increasing year after year, various types of keywords are related to the actual number of cases in these regions. In areas where the epidemic is declining yearly however, the correlation with the actual number of cases is low, and there is only a weak correlation between the therapeutic and diagnostic keywords. This may be due to the fact that the areas were previously hit hard by the epidemic. Since then, the daily related publicity and education efforts have been strengthened. In addition, the measures for intervening in the relevant departments have been strengthened so that the search index will inflate. Also, in the comparison of keyword search intensity in various provinces, the epidemic and search volume in Sichuan Province and Chongqing City are relatively high, but the search intensity of Internet users in the province is lower than those in Guangxi and Xinjiang where the epidemic situation is similar. It suggests that the local population in Sichuan Province maybe far less affected by medical institutions and media. Moreover, the government’s intervention policies and measures in this province may not be as strong as those in Guangxi and Xinjiang, resulting in a narrow demand for AIDS-related search by ordinary Internet users. Despite the lower keyword search strength, the BDI still has a high correlation with the AIDS epidemic in Sichuan province and can truly reflect the real case of AIDS^11^.

In summary, the search index of keywords cannot describe the epidemic situation of economically developed cities well. The overall search index in these regions may have a large deviation. Similarly, in some areas where the epidemic situation is stable and decreased, due to the influence of relevant policies and intervention measures, the inflation of search index will also affect the accuracy. However, if outbreaks are higher and the economy of the regions are underdeveloped, it can better describe the epidemic of AIDS.

The Com-ponent model, Delphi method, Asian Epidemic Model and Spec-trum model have been used extensively to estimate and predict the AIDS epidemics worldwide. These models however require a lot of biological indicators^15,16,27,28^, and a considerable amount of manpower and time. Based on China’s historical AIDS surveillance data, our study used time series analysis methods to establish ARIMA models and ARIMAX models for forecasting monthly new cases of HIV/AIDS in the country. We then used the two models to make short-term predictions of national AIDS epidemics. We found that using the ARIMAX model with BDI has smaller AIC value and MAPE value. The results suggested that using a multivariate ARIMAX model provides better prediction than a univariate model, and good predictability in terms of stability. As search queries can be processed quickly, integrating the ARIMA model with real time Baidu search engine query data may provide an early indicator for monitoring and detection of AIDS, improving the efficiency of the infectious disease surveillance system to better evaluate the epidemic situation, which in turn is crucial for the prevention and control of AIDS. This model, if it can be replicated under other conditions, may be available for the evaluation of new intervention measures against AIDS worldwide

As Baidu Inc is the largest search tool in China, its search queries could be a good representation of the needs of people’s lives, particularly in regions with high internet penetration rate. An early detection system could facilitate the timely intervention of the region and ease public misgivings about the health symptoms. However, due to the lack of manpower and material resources, the surveillance system in developing countries including China is limited^29–32^. Most HIV/AIDS cases in these areas were reported through a stepwise hierarchical reporting system in a sequence of town, county, city, province, and the national CDC. The proposed ARIMAX model integrating the timely search engine query data may provide opportunities to enhance the detection ability of the surveillance system for infectious diseases other than AIDS. A study by Yuan *et al*.^31^ used the time-series classification and regression models based on BDI to develop a predictive model for Influenza epidemics in China, which has shown stable predictive ability. And a previous research by Liu *et al*.^30^ introduced a method of analyzing large numbers of Baidu search queries to track dengue fever outbreaks in Guangzhou and Zhongshan, China. Incorporating BDI improved the fitness of the prediction model significantly in all these studies. Therefore, the Internet search data offers an effective instrument for government or public health agencies to monitor the HIV/AIDS epidemic early and takes steps accordingly.

However, there are some limitations of this current study. First of all, the search index of each keyword is easily influenced by the continuous change in search behavior of the individual, and Baidu Inc still has some related keywords not included, which may result in an underestimation of the correlation^8,22,33^. Therefore, the keywords used in this study only represent the search behavior of persons from 2011 to 2017, and cannot guarantee consistent and effective long-term prediction in the future. In addition, although the selected keywords capture the trend of outbreak data very well, there still may be some which increased as a result of social festivals, events and media reports. So, it is necessary to add or delete the corresponding keywords in the future and to ensure that there is a large correlation between the composite index and the actual number of cases. Thirdly, the use of Internet search data to assist epidemic surveillance depends on the amount of Internet access, which is uneven throughout China. The population sizes of different regions are also different. According to the latest Internet penetration rate of 55.8% released by the China Internet Network Information Center (CNNIC) in 2017, the rural Internet penetration rate reached only 35.4%. Finally, although we found that search engine data have spatial differences and there are also deviations in provincial network data. However, the selected provinces are still insufficient, as well as not found a pattern on different provincial relationships. Moreover, the ARIMAX model is based on nationwide data, does not take into account geographical disparities within the province. Further studies to account for the search index from a larger number of provinces and regions may be more effective.

In conclusion, the keywords for AIDS used in this study can provide some reference value for the collection, screening, analysis and forecasting process of search engine data. Moreover, the prediction model based on Baidu Index can accurately predict infectious disease outbreaks. Current monitoring and detection procedures for infectious disease epidemics are complicated and inefficient. Exploitation of the internet-based surveillance system by Baidu search index in disease surveillance is therefore timely. With the rapid development of the Internet services and search engine today, the combination of network data analysis may be considered as an adjunct for traditional monitoring of diseases. The real-time and low-cost Internet Big Data can improve the timeliness of monitoring and mitigate the low efficiency in the bureaucratic hierarchy, as well as help public health officials to identify and predict more accurately which people are at risk of potential HIV/AIDS spread to take effective interventions.

## Materials and Methods

### Data sources

Official case counts. This study used monthly aggregated HIV/AIDS new case counts from January 2011 to June 2017 (a total of 78 months) for China. The data is publicly available on China Centre for Disease Control and Prevention’s (China CDC) monthly status report of HIV/AIDS. In addition, it is typically released 1–2 week after the end of each month.

#### Baidu search index data

Daily search engine query data were obtained for the same period from Baidu index (http://index.baidu.com). The search’s database includes search query volumes for many keywords keyed in by Baidu search users. Moreover, Baidu index is available on a daily basis, at cities and towns, provinces and national level. Considering the HIV/AIDS category keywords are available in the Baidu’s search database from January 2011, therefore, we collected the data from January 2011 to June 2017. Since Baidu’s search index is available on a daily basis, so the index is converted to monthly counts for analysis.

An ethics committee is not required for this study because this study was based on official HIV/AIDS surveillance report data in China. There are no patients’ revealed information, thereby maintaining confidentiality.

### Keyword selection and screen

In the Baidu’s search index, the keywords have different search frequencies at different times and regions. Consequently, diverse search behavior can be reflected the attention of people from different periods. Although the significance of this is, however, there are no rules or standards for direction^31,34,35^. Some previous studies generally chose more of the associative names or clinical features of target illness as their crucial keywords^8,33,36^. Therefore, the primary keywords were deliberately picked to reflect terms most likely associated with HIV/AIDS. A Chinese website: https://ci.aizhan.com/ (here and later mentioned places are called keywords tool) was used for further obtaining correlative keywords and did some statistical analysis of collected numbers^31,34^. In addition, due to different people typing in entirely different words when searching the same information, especially when searching in Chinese language, where one meaning can be expressed in several ways, hence, some of the keywords represent the same meaning^37^. Other keywords were dug by using semantic correlation analysis from Microblog, Post Bar, and online Encyclopedia^31^. Finally, we initially acquired 100 related keywords about AIDS search behavior. However, some researches had indicated that more keywords do not necessarily assure better model fit and it’s not easily reproducible by research fellows with a finite resource^10,38^. Consequently, we collected a variety of HIV/AIDS core keywords and screened it following three steps:

- The core keywords should be principal factors that perhaps influence the genesis and development of the disease.
- We picked out each of the more than one million search frequent keywords by analyzing the total number of website inclusions and then 50 keywords remained.
- The selected keywords were divided into five categories, namely HIV/AIDS Etiology, General, Diagnosis, Symptom, and Treatment.

### Keyword analysis

In order to further screen out high relevance keywords and guarantee the number of keywords in our analysis. Therefore, in the monthly HIV/AIDS new case count and monthly keyword BDI Spearman’s correlations analysis (two-tailed test), we considered the Spearman’s rank correlation coefficients 0.6 as the threshold.

Furthermore, time-series cross-correlation analysis was used to examine the keywords whether having lag effects between the keywords and the HIV/AIDS new cases in different lag periods^32,37,39,40^. The lag value with the maximum correlation coefficient for each keyword was selected for inclusion in subsequent AIDS Search Index composition analysis. Then, considering the remaining keyword numbers and strength of the Spearman’s correlations coefficient, we deleted the words with maximum correlation coefficients less than 0.5 in each time lag and those correlations that were statistically insignificant. Finally, 17 keywords were detected with distinct non-lag in results of the cross-correlation analysis (Table 3).

### AIDS search index composition

The purpose of the composite index is to establish the relatively steady and correlative indicator for the HIV/AIDS case data based on the usable information. Following Spearman’s correlations analysis selection and cross-correlation analysis filtering, consequently, the final 17 keywords retained were applied for the composition of AIDS Search Index for each time lag. Additionally, we defined weights for each of the keywords by the strength of the Spearman’s correlation coefficient^31,35,37^. This method is commonly united with Analytic Hierarchy Process for better effects. Nevertheless, only using the correlation coefficient without adjustments appeared to be sufficient for this research^30,31,41^. The search index composition method and keywords weight counts are as Eqs 1 and 2.

1 $$Weigh{t}_{ki}={\rho }_{ki}/\sum _{i=1}^{n}{\rho }_{ki}$$

2 $$AIDS\,Search\,Inde{x}_{k}=\sum _{i=0}^{n}\,weigh{t}_{ki}\,keywor{d}_{ki}$$

In the above formulae, k denotes the potential time lag, n represents the number of keywords included at each time lag, $\rho \,$_ki_ is the Spearman’s rank correlation coefficient of included keyword (i) with specific time lag(k), keyword_ki_ and weight_ki_ denotes the i^th^ keyword monthly Baidu Index and the weight of it with specific time lag(k).

### Spatial difference analysis

In order to better observe the network search behavior of different types of people, it is necessary to carefully analyze the spatial distribution of each type of keyword.

Firstly, we selected Chongqing and Sichuan areas which with significantly increased AIDS epidemic, the Beijing and Shanghai regions where the epidemic progressed tardily, and the Xinjiang and Guangxi provinces with reduced epidemics as an example. Furthermore, according to the components of the national search index composition and the actual inclusion of Baidu search engine data in each region, a total of five categories of 17 keywords were selected for analysis. Thirdly, for better visualization of these data, we used the Search intensity indicator, which is calculated the total number of each keyword search indices and divided by number of Internet users in each provinces. Ultimately, we respectively analyzed the correlation between the actual number of cases in each province with the Internet users’ search intensity and the search index of keyword of five categories.

### Model construction

Time series models use the past tendency of variables so as to predict its future values. Most time series can be shown by Autoregressive Integrated Moving Average Model (ARIMA) model. The ARIMA model is also widely used to predict infectious diseases incidence through the use of historically surveillance cases data, such as malaria and hepatitis A, dengue fever and hepatitis incidence^19,42–44^. However, it is merely a univariate model, which also has limitations in predicting disease. The ARIMAX model is an extension of the Autoregressive Integrated Moving Average (ARIMA) model. More information (ie, Baidu search index and Google search queries) can be utilized by the ARIMAX model than the ARIMA model, it makes use of multiple regression analysis and time series analysis that could improve the forecasting ability^42,45^. Therefore, we used the ARIMA model that combines Baidu Composite Index (AIDS search index) and can examine the relationship between keywords and AIDS. The following equation was used to obtain the predicting disease series:

3 $${y}_{t}=\mu +\sum _{i=1}^{k}\frac{{\Theta }_{i}(B)}{{\Phi }_{i}(B)}{B}^{{l}^{i}}{x}_{it}+{r}_{i}$$

4 $${r}_{i}=\frac{{\Theta }_{i}(B)}{{\Phi }_{i}(B)}{a}_{t}$$

In the formula $\,{\Theta }_{i}(B)$, ${\Phi }_{i}(B)\,$and ${l}^{i}\,$were the autoregressive coefficients polynomial, moving average coefficient polynomial and a lag operator of the *i*th input variables respectively; ${x}_{it}$ denotes external variables AIDS Search Index_k_; ${r}_{i}$ was the regression residual sequence; and ${\Theta }_{i}(B)\,$and ${\Phi }_{i}(B)\,$are respectively the residual autoregressive coefficient polynomial and the residual moving average coefficient polynomial; ${r}_{i}$ is the residual sequence, and ${a}_{t}$ is the white noise sequence with zero mean. ${y}_{t}$ was the dependent variable^46,47^.

The predicting precision of the model was checked by the goodness of fit between the observed and predicted of HIV/AIDS report cases using the mean absolute percentage error (MAPE)^48–50^, equation was as follow:

5 $${\rm{MAPE}}=\sum _{t=1}^{n}|\frac{observe{d}_{t}-predicte{d}_{t}}{observe{d}_{t}}|\times \frac{100}{n}$$

It means that predicted cases from the models have a better accuracy for the reported case when the MAPE values are small.

The modeling process of ARIMA and ARIMAX was analyzed by SAS 9.4 statistical software and the rest of the statistical analysis were performed using IBM SPSS 19.0. Furthermore, each variable with *P*<0.05 were considered to be significant in the analysis and construction process.

## Electronic supplementary material

Supplementary Figure S1-S4
