# Supplementary material for: Using Baidu Search Engine to Monitor AIDS Epidemics Inform for Targeted intervention of HIV/AIDS in China
Source: Sci Rep. 2019 Jan 23;9:320. doi: 10.1038/s41598-018-35685-w (PMC6344537; doi:10.1038/s41598-018-35685-w)
Supplement: Supplementary file 1 — Supplementary Figure S1-S4 [file 41598_2018_35685_MOESM1_ESM.pdf]

Using Baidu Search Engine to Monitor AIDS Epidemics Inform for Targeted intervention of HIV/AIDS in China

Kang Li[1,2], Meiliang Liu[1], Yi Feng[2], Chuanyi Ning[1], Weidong Ou[1,2], Jia Sun[2], Wudi Wei[1], Hao Liang[1*] &Yiming Shao[1,2*]

[1]Guangxi Key Laboratory of AIDS Prevention and Treatment & Guangxi Universities Key Laboratory of Prevention and Control of Highly Prevalent Disease, School of Public Health, Guangxi Medical University, Nanning, Guangxi, China
[2]State Key Laboratory for Infectious Disease Prevention and Control, National Center for AIDS/STD Control and Prevention, Chinese Center for Disease Control and Prevention, Beijing, China

Supplementary Information

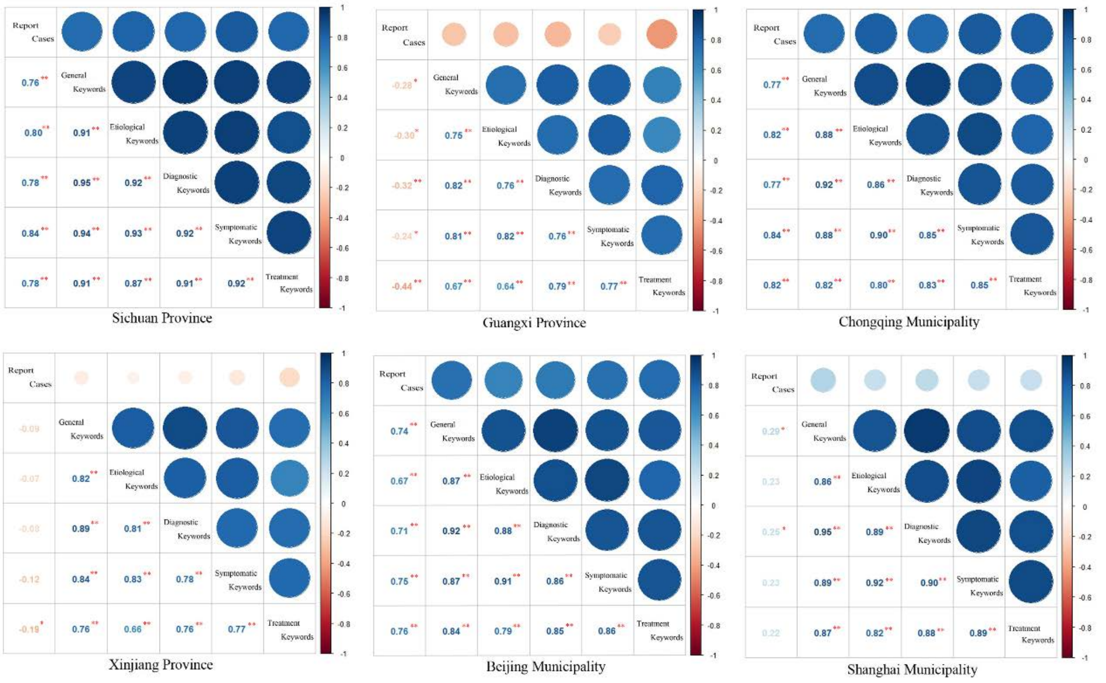

Figure S1. Number of HIV/AIDS cases and five types of keywords Search query data pairwise correlation matrix in six provinces from 2011 to 2016. $^{*}P$<0.05, $^{**}P$<0.001

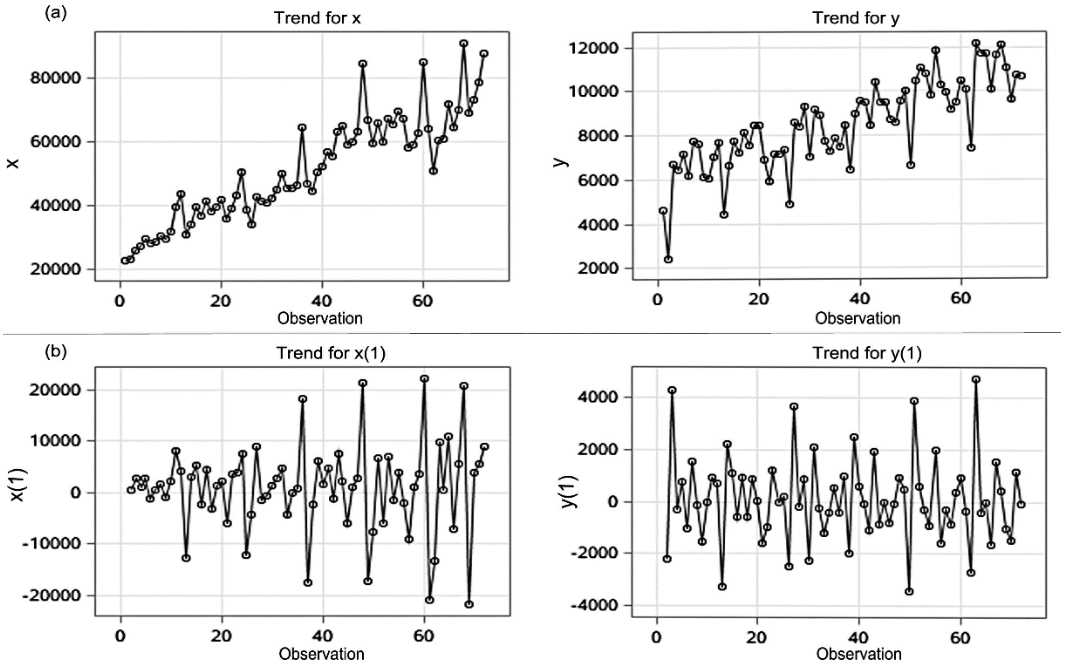

Figure S2. Time series diagram of data sequence. The sequences x and

y respectively represent the national Baidu composite index and the number of report cases of HIV/AIDS monthly from January 2011 to December 2016. (a)The original data sequence; (b) The First-order differential data sequence.

(a)

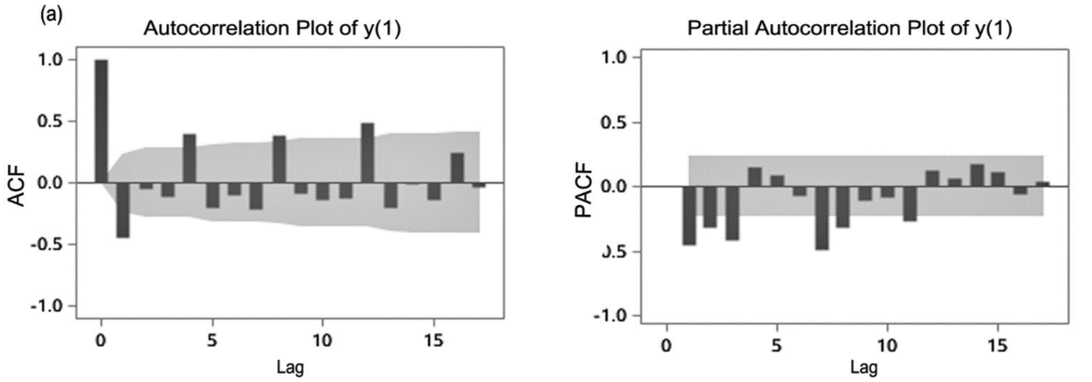

(b)

Autocorrelation Check of Residuals									
To Lag	Chi-Square	DF	Pr > ChiSq	Autocorrelations					
6	3.93	3	0.2688	0.008	-0.050	0.041	0.115	-0.160	-0.086
12	11.67	9	0.2328	-0.009	0.000	0.028	-0.087	0.154	-0.238
18	16.06	15	0.3778	0.037	0.008	-0.035	-0.116	0.052	-0.164
24	27.28	21	0.1618	-0.191	0.194	0.022	-0.022	-0.048	0.176

Figure S3. Autocorrelation function (ACF), partial ACF (PACF) and Autocorrelation check of residuals plots of differencing HIV/AIDS report cases. The X-axis gives the number of lags in weeks, the y-axis is the value of the correlation coefficient, and the gray zone illustrate 95% confidence interval.

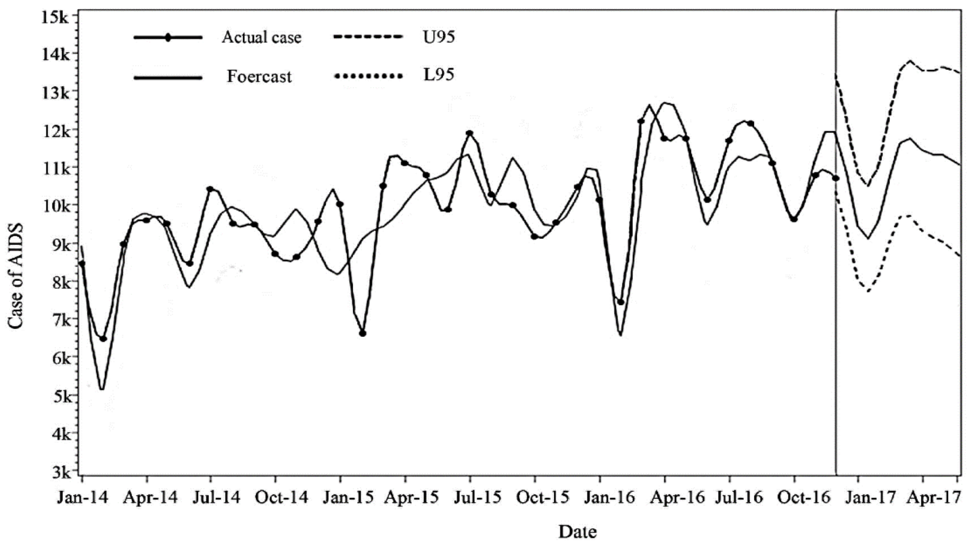

Figure S4. The predicting curves of HIV/AIDS report cases in China.

L = The lower limit of 95% confidence interval; U = The upper limit of 95% confidence interval.
